# Constructing optimal experience for the hospitalized newborn through neuro-based music therapy

**DOI:** 10.3389/fnhum.2015.00487

**Published:** 2015-09-03

**Authors:** Helen Shoemark, Deanna Hanson-Abromeit, Lauren Stewart

**Affiliations:** ^1^Music Therapy, Temple UniversityPhiladelphia, PA, USA; ^2^Sensory Experience in Early Development, Murdoch Childrens Research InstituteMelbourne, VIC, Australia; ^3^School of Music, University of KansasLawrence, KS, USA; ^4^Department of Psychology, Goldsmiths, University of LondonNew Cross London, UK; ^5^Center for Music in the Brain, Department of Clinical Medicine, Aarhus University and The Royal Academy of Music Aarhus/AalborgAarhus, Denmark

**Keywords:** neonatal intensive care unit, music therapy, preterm infant, auditory environment, stimulation, family centered practice

## Abstract

Music-based intervention for hospitalized newborn infants has traditionally been based in a biomedical model, with physiological stability as the prime objective. More recent applications are grounded in other theories, including attachment, trauma and neurological models in which infant, parent and the dyadic interaction may be viewed as a dynamic system bound by the common context of the neonatal intensive care unit (NICU). The immature state of the preterm infant’s auditory processing system requires a careful and individualized approach for the introduction of purposeful auditory experience intended to support development. The infant’s experience of an unpredictable auditory environment is further compromised by a potential lack of meaningful auditory stimulation. Parents often feel disconnected from their own capacities to nurture their infant with potentially life-long implications for the infant’s neurobehavioral and psychological well-being. This perspectives paper will outline some neurological considerations for auditory processing in the premature infant to frame a premise for music-based interventions. A hypothetical clinical case will illustrate the application of music by a music therapist with an infant and family in NICU.

## Introduction

The premature infant brain exhibits a distinctive type of white matter injury, affecting the cerebral white matter, thalamus, basal ganglia, cerebral cortex, brainstem and cerebellum (Volpe, 2009). Regional brain volumes acquired from children, aged 8 years, who were born preterm, show differences across a number of cortical and subcortical brain regions (sensorimotor, premotor, mid-termporal, occipto-parietal, basal ganglia, cerebellum, hippocampus, corpus callosum) several of which correlate with poorer cognitive outcomes (full-scale, verbal and performance IQ scores; Peterson et al., 2000). While such an abnormal neurological profile will have profound consequences for subsequent neuronal and cognitive development, this may be mitigated, at least in part, by providing the most conducive environment for promoting neurological development, given the established role of experience and environment in shaping brain structure and function (van Praag et al., 2000). The present article argues that music-based interventions, grounded in multi-faceted theories, can play a role in optimizing the experience and environment of the preterm infant, particularly when implemented by a music therapist and working alongside parents.

The environment in which a premature infant is cared for is highly atypical when compared to that of a term infant born without medical complications. Certain aspects of the “normal” environment are missing: opportunities for predictable sensory stimulation as well as for social interaction (including directed linguistic input). Other aspects are uniquely present in the neonatal intensive care unit (NICU): high levels of unpredictable noise, impinging upon an auditory system that is normally “protected” by the attentuating maternal tissues, as well as frequent invasive procedures. Infants are frequently startled, sleep is disrupted, and physiological profile is unstable (Graven, 2000). While the NICU environment is doubtless key to survival, it nonetheless constitutes a considerable source of stress for the preterm infant (Newnham et al., 2009). While we might assume that even the preterm brain will exhibit some resilience to this, owing to the presence of the fast acting sympathetic-adrenal system, as well as the slower acting hypothamalic-pituitary-adrenal axis (HPA), current research suggests that the impact of prolonged or excessive stress has the potential to result in a dysregulated response to future stressors, as well maladaptive changes in the hippocampus, prefrontal cortex and amygdala—areas which are important for learning, decision making and emotion regulation (Radley and Morrison, 2005).

The removal of noise alone is now considered an inadequate response to the traditional problem of inappropriate auditory stimulation in the NICU (Jobe, 2014). While attention to reducing noise is necessary, equal attention must be given to the creation of meaningful auditory stimulation (Rand and Lahav, 2013). Compared to other environmental sounds, musical sound waves have organized structural characteristics of pitch, dynamics, timbre and harmony. Thus, once noise has been reduced, carefully selected music can be used as an environmental stimulus within the recommended decibel levels for stimulation (45–50 dBA). For the infant to detect the music it will need to be sufficiently loud or have frequencies or timbres which distinguish it from the ambient environment (Dearn and Shoemark, 2014). Guiding principles to consider in terms of musical characteristics include predictable patterns in rhythm, melody, and phrasing (Trehub and Trainor, 1990; Gray and Philbin, 2004), sparse gradual changes in tempo in a lullaby style (Trainor, 1996; Rock et al., 1999), consonance (Trainor et al., 2002), smooth melodic contours (Unyk et al., 1992) and an absence of harmonies (Janata et al., 2002; Gray and Philbin, 2004; Siddiqui et al., 2008). While full-term and older infants attend to higher pitches (Trehub and Trainor, 1990; Trehub, 2001), the premature infant’s auditory development may be better suited to lower pitches (Graven and Browne, 2008). However, it is essential for the characteristics of the music to be explicitly tailored and adjusted to the infants needs (Hanson-Abromeit, 2015) as well as to acknowledge the preferences of the family to ensure a culturally acceptable and useful stimulus formulated for their family functioning. Thus the individualized application of music requires a relationship between music therapist, infant and family, which differentiates it from other music-based approaches where music may be a more standardized stimulus.

In addition to constituting a source of chronic stress for the premature infant, the unpredictable and noisy environment of the NICU has a detrimental impact on the infant’s sleep patterns (Kuhn et al., 2012) and interrupts the transitioning between different physiological states which is important for the newborn infant. A study by Weisman et al. (2011) showed that infants whose sleep-state transitions were mainly characterized by shifts between quiet sleep and wakefulness exhibited the best development, including greater neonatal neuromaturation, less negative emotionality, better cognitive development, and better verbal, symbolic, and executive competences at 5 years. Music can be useful in helping the infant transition effectively between physiological states—for instance from quiet wakefulness to sleep. Early research in this field from Olischar et al. (2011) reported a trend towards more mature sleep-wake cycling in healthy infants at 32 weeks or more gestational age when exposed to a recording of a lullaby. The ability to transition effectively between sleep states, wakefulness, and stress states is also a significant benefit in relation to the many medical and diagnostic procedures that occur in the NICU. Many of these involve pharmacological sedation which can result in adverse reactions, including nausea and vomiting, respiratory depression and cardiac arrhythmias (Harvey, 1985; Greenberg et al., 1993; Paret et al., 1996). There is some evidence to suggest that the use of music, either exclusively, or in combination with a lower dose of pharmacological sedative, can provide an effective and safe alternative (Loewy et al., 2005; Schwilling et al., 2015).

While some aspects of stress in the NICU relate to the presence of noxious auditory stimuli, other aspects relate to the absence of a consistent and appropriate response from a primary caregiver, particularly given the infant’s state of physiological dysregulation. This physical separation is also a source of stress for the parents, for whom the typical biological processes of feeding, nurturing and bonding with their infant are curtailed owing to the complex medical needs of the child. Problems of attachment are common, with parents enduring significant experiences of trauma (Coppola et al., 2007) putting at risk their own sensitivity to their baby, and often developing an overall hypervigilance about their baby’s medical status. A secure attachment to a caregiver is important for the cognitive development of self vs. other representations, as well as for the providing a template for the formation of interpersonal relationships throughout the lifespan (Borghini et al., 2006; Vrticka and Vuilleumier, 2012). One strong advantage of using music in the NICU concerns its potential for a family-based approach to care, which can promote mutual regulation of the parent-infant dyad, thus building attachment and empowering parents to be involved in the care of their child (Shoemark and Dearn, 2008), as well as providing them with a tool they can use beyond the NICU, bearing in mind that the neurodevelopmental challenges that result from prematurity can last well beyond this early period.

The use of maternal voice can hold special promise for promoting normal attachment (Milligan et al., 2003): fetuses and newborn infants can recognize and orient to their mother’s voice, in comparison with a female stranger (DeCasper and Fifer, 1980); mother’s voice has been found to increase preterm infant oxygen saturation levels, reducing the occurrence of critical events and inducing quiet alert states (Filippa et al., 2013) and benefitting parental stress levels (Loewy et al., 2013). When singing to infants, caregivers adopt a particular “infant-directed” style, characterized by higher pitch, slower tempo and distinctive timbre (Trehub et al., 1997). This style of singing is seen across cultures and attracts and maintains the infant’s attention (Trehub and Schellenberg, 1995; Van Puyvelde et al., 2015), and ameliorates distress in older infants more effectively than speech or touching (Trehub et al., 2015), all allowing opportunities for communication and interaction, which are the basis for building secure attachment. In the NICU maternal voice is a means of social communication and emotional and physiological regulation, when physical closeness between parents and infant is often precluded.

Live aspects of maternal singing allow the care-giver to intuitively adapt aspects of their vocalizations to match or alter their infant’s state (infant-directed singing), encouraging a reciprocal relationship between the dyad (Hanson-Abromeit, 2003). Singing by an attuned care-giver has been shown to produce a significant benefit for neurobehavioral development in a small group of medically complex newborn infants (Malloch et al., 2012). Integrating parents’ cultural systems within the context of infant-directed singing may encourage parent attunement to their infant’s cues and support attachment through responsiveness (Loewy, 2015) particularly if it is modified with consideration for the infant’s neurological functioning (Stewart, 2009). The music therapist can use principles of maternal self-efficacy (Črnčec et al., 2008) to help the parent construct their singing until it begins to feel more natural encouraging them to sing directly to their infant (Mondanaro, 2010; Shoemark, 2013).

The case study below is a hypothetical case constructed to specifically illustrate the principles outlined above in a real-world application of music-based interventions across a premature infant’s hospitalization (Shoemark, 2014).

## Case Study

Cheryl and Danny’s son Jake was born at 25 weeks gestation. In the first days, Cheryl felt powerless, and simply sat watching his tiny form amidst the technology. She would occasionally whisper through the porthole and hold her hand just above his fragile skin to offer him human contact. The music therapy referral was made to establish the parents’ sense of self-efficacy as his parents and maintain a strong sense of their unique place as his parents until Jake was available for age-appropriate neurodevelopmental opportunities.

The music therapist explained to Jake’s parents that it would be some weeks before Jake could actively respond to their voices but that exposure each day would be helpful (Doheny et al., 2012). She encouraged them to voice their words rather than whisper as this would have more value to him (Spence and Freeman, 1996) and to use the replicable pattern of song and rhymes which would eventually become familiar to him and mark their presence. Each morning his father would gently sing the opening line of his college chant, with its distinctive low-pitched and descending interval “Rock chalk, jay hawk, K-U …”. After a couple of weeks, Jake began to open his eyes when Danny sang. The parents later reported that this recognition of their experience together had been pivotal in their evolving attachment.

Around 36 weeks, Jake was having trouble getting to, and staying asleep. The nurse made a referral to the music therapist for recorded music to support state transition. Before assessing directly, the music therapist confirmed this need with Jake’s mother (acknowledging her pivotal role) who agreed and added that Jake often startled at noise. So for a period after each feed and cares, the music therapist measured the ongoing ambient sound (OAS) level and noise events that were 12 dBA or more. This revealed that: (a) the OAS level was mostly at 55 dBA but there were frequent noise events from the nurses’ station outside his room (phone receiver put down, calling out, chair scrapes); and (b) the noise events were detected by Jake (facial flinches, jittery limb movement, repeated disruption to evolving sleep), causing sustained arousal. Two strategies were implemented: Jake’s bed was removed from the immediate vicinity of the nurse’s station resulting in a reduction in the ambient sound level by 5 dBA (which is significant in perceived loudness), and the number of noise events was reduced by 60%. Secondly, the music therapist prepared a 20 min recorded music playlist played at just over 50 dBA which provided a predictable ambient auditory field over which only a few noise events were notable. Mother and nurses reported that Jake’s response was positive with regular transition to sleep occurring within 48 h.

Cheryl talked to Jake each day, discovering for herself that this simple act could settle his breathing rate after the nurses took physiological observations. The music therapist introduced Cheryl to contingent singing, using her voice to consciously support Jake’s state regulation and learning about his unique cues to ensure the interaction was within Jake’s thresholds for stimulation (Shoemark, 2012). As he became more socially available she agreed with the music therapist that a repertoire of playsongs and lullabies would be a useful extension for their interaction. In discussion with Cheryl’s mother on her next visit, the music therapist explored the family’s relationship with music, to explicate the potential of song as source of joint attention and intersubjectivity (Malloch et al., 2012). She revealed that singing was always a part of Cheryl’s early years, establishing an inter-generational role of music as part of nurturing in this family. As a small child, Cheryl had been transfixed by the Bryan Adams movie theme “Everything I do, I do it for you” and would giggle and move to Whitney Houston’s “I will always love you”. They were repeated daily. Through this recollection the mother and grand-mother shared a moment of emotional connection which strengthened the mother’s relationship with music (Tronick, 1998). With a guitar accompaniment, the music therapist quietly sang the chorus “And I-I will always love you …” to bridge the introduction of these songs into the NICU for the two women. They reminisced about how they copied Houston’s extended “I-I-I” in the chorus, thus engaging their own musical heritage (Loewy et al., 2013). The music therapist redirected the song towards Jake asleep in his humidicrib. She encouraged mother and grand-mother to sing or hum so Jake could hear their voices. Cheryl’s mother was able to sing and Cheryl leaned in through the porthole and hummed as she put her hand on Jake’s torso. The song was thus coupled with the more familiar touch, providing a smooth introduction to this new experience for Jake.

## Conclusion

This perspectives paper has considered some of the experiential and environmental issues confronting the preterm hospitalized infant. Given the significant impact of experience and environment on brain plasticity, offering an enriched environment via individualized music therapy, is suggested to provide a more optimal context to facilitate neurological development. As the application of music-based interventions continues to grow in the NICU it is important to consider a theoretical framework grounded in neurological mechanisms, theories of infant-directed singing, development and attachment, as well as the clinical expertise of the music therapist. Music therapists are uniquely placed to adapt the soundscape of the NICU and encourage parental vocal communication in order to promote sensory-system maturation, to reduce the impact of environmental stresses, to facilitate transitioning between different physiological states and to foster attachment. Such music-based interventions are an important and viable option to accommodate the developing neurological status of the premature infant and have the potential to benefit both short and long-term outcomes in the infant and parent.

## Conflict of Interest Statement

The authors declare that the research was conducted in the absence of any commercial or financial relationships that could be construed as a potential conflict of interest.
